# 巴利昔单抗治疗糖皮质激素耐药/依赖急性移植物抗宿主病87例疗效分析

**DOI:** 10.3760/cma.j.issn.0253-2727.2022.02.006

**Published:** 2022-02

**Authors:** 振新 贺, 荣莉 张, 卫华 翟, 巧玲 马, 爱明 庞, 栋林 杨, 祎 何, 嘉璘 魏, 欣 陈, 尔烈 姜, 四洲 冯, 明哲 韩

**Affiliations:** 中国医学科学院血液病医院（中国医学科学院血液学研究所）造血干细胞移植中心，实验血液学国家重点实验室，国家血液系统疾病临床医学研究中心，天津 300020 State Key Laboratory of Experimental Hematology, National Clinical Research Center for Blood Diseases, Institute of Hematology & Blood Diseases Hospital, Chinese Academy of Medical Sciences & Peking Union Medical College, Tianjin 300020, China

**Keywords:** 巴利昔单抗, 急性移植物抗宿主病, 造血干细胞移植, Basiliximab, Graft-versus-host disease, Hematopoietic stem cell transplantation

## Abstract

**目的:**

分析巴利昔单抗治疗异基因造血干细胞移植后糖皮质激素耐药/依赖急性移植物抗宿主病（aGVHD）患者的疗效。

**方法:**

对2015年1月至2018年12月期间于中国医学科学院血液病医院行异基因造血干细胞移植后发生皮肤、肠道、肝脏aGVHD并应用巴利昔单抗治疗的87例血液病患者进行回顾性分析。巴利昔单抗用药方案：成人及体重≥35 kg儿童每次20 mg，体重<35 kg儿童每次10 mg，第1、4、8天各给药1次，以后每周用药1次，在巴利昔单抗治疗后第7、14、21、28、42、56天分别进行疗效评估。

**结果:**

①87例患者中，男51例（58.6％），女36例（41.4％），中位年龄34（4～63）岁，经典型aGVHD 54例，迟发型aGVHD 33例，糖皮质激素耐药49例，糖皮质激素依赖38例。②35例（40.2％）获得完全缓解（CR），23例（26.4％）获得部分缓解（PR），29例患者未缓解（NR），总有效率（ORR）为66.7％（58/87）。③经典型aGVHD组、迟发型aGVHD组ORR分别为77.8％（42/54）、48.5％（16/33）。④中位随访154（4～1813）d，全部87例患者移植后6个月总生存（OS）率为44.8％（95％*CI* 39.5％～50.1％），移植后1年OS率为39.4％（95％*CI* 34.2％～44.3％）。⑤巴利昔单抗治疗后CR组（35例）、PR组（23例）、NR组（29例）移植后6个月OS率分别为80.0％（95％*CI* 73.2％～86.8％）、39.1％（95％*CI* 28.9％～49.3％）、6.9％（95％*CI* 2.2％～11.6％）（*χ*^2^＝34.679，*P*<0.001），移植后1年OS率分别为74.3％（95％*CI* 66.9％～81.7％）、30.4％（95％*CI* 20.8％～40.0％）、3.4％（95％*CI* 0％～6.8％）（*χ*^2^＝43.339，*P*<0.001）。经典型aGVHD组、迟发型aGVHD组移植后6个月OS率分别为57.4％（95％*CI* 50.7％～64.1％）、24.2％（95％*CI* 16.7％～31.7％）（*χ*^2^＝9.109，*P*＝0.004），移植后1年OS率分别为51.9％（95％*CI* 45.1％～58.7％）、18.2％（95％*CI* 11.5％～24.9％）（*χ*^2^＝9.753，*P*＝0.003）。⑥单因素及多因素分析结果显示，迟发型aGVHD（*OR*＝3.121，95％*CI* 1.770～5.503，*P*<0.001）、用药前明尼苏达积分高危组（*OR*＝3.591，95％*CI* 1.931～6.679，*P*<0.001）、用药前存在活动性感染（*OR*＝1.881，95％*CI* 1.029～3.438，*P*＝0.040）、伴有非GVHD所致重要脏器功能受损（*OR*＝3.100，95％*CI* 1.570～6.121，*P*＝0.001）是影响巴利昔单抗疗效的独立危险因素。

**结论:**

巴利昔单抗对于糖皮质激素耐药/依赖aGVHD具有良好的疗效和安全性；迟发型、用药前明尼苏达积分高危组、存在感染或重要脏器功能受损患者疗效不佳。

## Abstract (en)

**Objective:**

To evaluate the efficacy and prognosis of basiliximab in the treatment of steroid-refractory or steroid-dependent acute graft-versus-host disease (SR/SD-aGVHD) after allogeneic hematopoietic stem cell transplantation (allo-HSCT).

**Methods:**

Clinical data of 87 patients with SR/SD-aGVHD in the skin, intestine, and liver after allo-HSCT at the Institute of Hematology & Blood Diseases Hospital Transplantation Center from January 2015 to December 2018 were retrospectively analyzed. The administration plan of basiliximab was as follows: 20 mg for adults and children weighing ≥35 kg and 10 mg for children weighing <35 kg. The drug was administered once on the 1st, 4th, and 8th days, respectively, and then once weekly. The efficacy was evaluated on the 7th, 14th, 21st, and 28th days after basiliximab treatment.

**Results:**

①There were 51 males（58.6％）and 36 females（41.4％）, with a median（range）age of 34（4-63）years. There were 54 cases of classic aGVHD, 33 of late aGVHD, 49 of steroid-refractory aGVHD, and 38 of steroid-dependent aGVHD. ②Thirty-five patients（40.2％）achieved complete remission（CR）, 23（26.4％）achieved partial remission（PR）, and 29 had no remission（NR）. The total effective rate［overall response rate（ORR）］was 66.7％（58/87）. ③The ORR of the classic and late aGVHD groups was 77.8％（42/54）and 48.5％（16/33）, respectively. ④The median（range）follow-up time was 154（4–1813）days, the 6-month overall survival（OS）rate of the 87 patients was 44.8％（95％ *CI* 39.5％-50.1％）and the 1-year OS was 39.4％（95％ *CI* 34.2％–44.3％）. ⑤After treatment with basiliximab, the 6-month OS in the CR（35 cases）, PR（23 cases）, and NR（29 cases）groups was 80.0％（95％ *CI* 73.2％–86.8％）, 39.1％（95％ *CI* 28.9％–49.3％）, and 6.9％（95％ *CI* 2.2％–11.6％）, respectively（*χ*^2^＝34.679, *P*<0.001）, and the 1-year OS was 74.3％（95％ *CI* 66.9％–81.7％）, 30.4％（95％ *CI* 20.8％–40.0％）, and 3.4％（95％ *CI* 0％–6.8％）, respectively（*χ*^2^＝43.339, *P*<0.001）. The OS of the classic and late aGVHD groups was 57.4％（95％ *CI* 50.7％–64.1％）and 24.2％（95％ *CI* 16.7％–31.7％）, respectively（*χ*^2^＝9.109, *P*＝0.004）, and the 1-year OS was 51.9％（95％ *CI* 45.1％–58.7％）and 18.2％（95％ *CI* 11.5％–24.9％）, respectively（*χ*^2^＝9.753, *P*＝0.003）. ⑥Univariate and multivariate analyses showed that late aGVHD（*OR*＝3.121, 95％ *CI* 1.770–5.503, *P*<0.001）, Minnesota score high-risk group before medication（*OR*＝3.591, 95％ *CI* 1.931–6.679, *P*<0.001）, active infection before medication（*OR*＝1.881, 95％ *CI* 1.029–3.438, *P*＝0.040）, and impairment of important organ function caused by non-GVHD（*OR*＝3.100, 95％ *CI* 1.570–6.121, *P*＝0.001）were independent risk factors affecting the efficacy of basiliximab.

**Conclusion:**

Basiliximab has good efficacy and safety for SR/SD-aGVHD, but not in patients with late aGVHD, high-risk group of Minnesota score, and infection or impaired function of important organs.

异基因造血干细胞移植（allo-HSCT）是治疗多种血液疾病最有效方法之一，急性移植物抗宿主病（aGVHD）是其主要并发症和死亡原因[1]–[2]。目前国内报道中度至重度aGVHD的发生率为13％～47％，糖皮质激素一线治疗的总体有效率约为50％[1]。巴利昔单抗（IL-2RA、CD25单抗）是一种鼠/人嵌合的单克隆抗体（IgG1K），通过特异性与T细胞表面IL-2受体的α链（CD25抗原）结合，从而阻断IL-2介导的T淋巴细胞活化与增殖，是目前我国广泛采用的二线治疗手段之一，文献报道巴利昔单抗对成人糖皮质激素耐药/依赖aGVHD患者的总有效率达78.7％～86.8％，完全缓解（CR）率达60.9％～69.8％[3]–[5]；对儿童单倍型造血干细胞移植（haplo-HSCT）后糖皮质激素耐药/依赖aGVHD的总有效率为85％，CR率为74％[6]。本研究纳入2015年1月至2018年12月在中国医学科学院血液病医院移植中心接受巴利昔单抗治疗的87例allo-HSCT后糖皮质激素耐药/依赖aGVHD患者，对巴利昔单抗治疗糖皮质激素耐药/依赖aGVHD的疗效及影响因素进行回顾性分析。

## 病例与方法

1. 病例纳入标准：本回顾性研究纳入符合以下条件的患者：①2015年1月至2018年12月在中国医学科学院血液病医院接受allo-HSCT后发生皮肤、肠道、肝脏aGVHD；②在常规应用环孢素A（CsA）、他克莫司（FK506）或霉酚酸酯（MMF）治疗基础上加用一线治疗（甲泼尼龙1～2 mg·kg^−1^·d^−1^）出现的糖皮质激素耐药/依赖aGVHD患者；③二线治疗方案首选巴利昔单抗或其他二线方案治疗无效后加用巴利昔单抗；④巴利昔单抗使用次数≥2次。

2. 诊断与分级：aGVHD及糖皮质激素耐药/依赖aGVHD诊断参照国内专家共识[1]推荐标准。经典型aGVHD指发生于移植后100 d（+100 d）内，且主要表现为皮肤、胃肠道和肝脏三个器官的炎性反应；迟发型（晚发型）aGVHD指具备经典型aGVHD的临床表现但发生于+100 d后的GVHD。aGVHD严重程度评估采用国际联盟（MAGIC）分级标准[7]。明尼苏达危险度积分[8]主要通过对aGVHD受累器官程度及数量进行分类组合量化评估，将aGVHD分为标危和高危组，可预测aGVHD对糖皮质激素的反应[9]，同时可以预测GVHD的生存和移植相关死亡。本研究也采用明尼苏达积分对纳入病例进行分析。

3. 预处理：所有血液肿瘤患者均采用清髓预处理方案。急性髓系白血病（AML）、慢性髓性白血病（CML）、骨髓增生异常综合征（MDS）等髓系肿瘤进行同胞全相合移植患者应用改良白消安（Bu）+环磷酰胺（Cy）预处理；髓系肿瘤行无关供者移植及haplo-HSCT患者应用改良Bu+Cy+抗胸腺细胞球蛋白（ATG）预处理方案；重型再生障碍性贫血（SAA）患者应用氟达拉滨（Flu）+Cy+ATG预处理方案；髓外侵犯或急性淋巴细胞白血病（ALL）行同胞全相合供者移植患者应用改良全身照射（TBI）+Cy预处理方案，髓外侵犯或ALL行无关供者移植及haplo-HSCT患者应用改良TBI+Cy+ATG预处理。

4. GVHD预防：应用CsA或他克莫司及短疗程甲氨蝶呤（MTX）预防GVHD。haplo-HSCT和无关供者移植加用MMF。CsA：起始剂量1 mg/kg，每12 h 1次，持续静脉输注，−8 d开始给药，消化道症状消失后改为口服给药。他克莫司：起始剂量0.03 mg·kg^−1^·d^−1^，−1 d开始给药，其他原则同CsA。MTX：+1 d予以15 mg/m^2^，+3 d、+6 d、+11 d各10 mg/m^2^，静脉注射给药（同胞全合移植取消+11 d用药）。MMF：成人或体重≥35 kg儿童1000 mg/d，体重<35 kg儿童30 mg·kg^−1^·d^−1^，每日剂量分2次服用，−9 d开始使用。

5. aGVHD治疗：基础治疗采用CsA或他克莫司±MMF。一线治疗应用甲泼尼龙1～2 mg·kg^−1^·d^−1^（分2次给药）。二线治疗方案包括巴利昔单抗、英夫利昔单抗、依那西普、芦可替尼、Cy、间充质干细胞。

巴利昔单抗具体用法：成人及体重≥35 kg儿童每次20 mg，体重<35 kg儿童每次10 mg，第1、4、8天各给药1次，以后每周用药1次，疗程根据具体病情决定。

6. 疗效判定：CR：aGVHD的表现完全消失且维持至少1周；部分缓解（PR）：至少1个器官出现改善（评分降低至少1级），其他器官情况稳定，维持时间≥1周；未缓解（NR）：原有器官症状无改善或至少1个器官出现进展（评分升高1级）。总反应率（ORR）为CR及PR患者在全部患者中的占比（％）。

7. 随访：随访资料来自门诊/住院病历及电话随访。末次随访日期为2020年6月30日。总生存（OS）时间：从巴利昔单抗治疗开始至因任何原因导致死亡的时间，对于死亡之前失访的患者，将末次随访时间计算为死亡时间。

8. 统计学处理：采用SPSS 26.0和R软件进行数据分析。分类变量采用*χ*^2^检验或Fisher精确检验。生存分析采用Kaplan-Meier曲线法。累积复发率、非复发死亡率采用竞争分析模型分析。多因素分析采用Cox比例风险回归模型，双侧*P*<0.05为差异有统计学意义。应用Graphpad Prism 8软件绘图。

## 结果

1. 患者特征：本研究共纳入87例患者，其中男51例（58.6％），女36例（41.4％）。中位年龄34（4～63）岁。经典型aGVHD患者54例，迟发型aGVHD患者33例（形态学或分子遗传学复发患者输注供者淋巴细胞诱发27例，+100 d后复发5例，重叠综合征1例）。经典型aGVHD组发生aGVHD中位时间为移植后29（9～74）d，迟发型aGVHD组发病距移植的时间差异较大，未作统计。输注单个核细胞中位数为8.43（4.89～17.20）×10^8^/kg，CD34^+^细胞中位数为3.12（2.00～6.89）×10^6^/kg。中位中性粒细胞植入时间为13（10～75）d，中位血小板植入时间17（10～105）d。中位巴利昔单抗使用次数为4（2～6）次。患者基线临床资料见表1。

**表1:** 87例接受巴利昔单抗治疗糖皮质激素耐药/依赖急性移植物抗宿主病（aGVHD）患者的基线资料［例（％）］

临床特征	结果
年龄	
≤40岁	55（63.2）
>40岁	32（36.8）
性别	
男	51（58.6）
女	36（41.4）
诊断	
AML	27（31.1）
ALL	14（16.1）
CML	3（3.4）
MDS	23（26.4）
SAA	10（11.5）
其他	10（11.5）
恶性血液病移植前状态	
CR1	31（35.6）
非CR1	43（49.4）
HCT-CI评分	
低危组	62（70.2）
中、高危组	25（29.8）
造血干细胞来源	
外周血	69（79.3）
外周血+骨髓	17（19.5）
骨髓	1（1.2）
供受者性别关系	
女供男	17（19.5）
非女供男	70（80.5）
供者类型	
同胞全相合	33（37.9）
单倍型	50（57.5）
无关供者	4（4.6）
中性粒细胞植活	87（100.0）
血小板植活	83（95.4）
aGVHD类型	
经典型	54（62.1）
迟发型	33（37.9）
受累器官	
单纯皮肤	3（3.5）
单纯肠道	20（23.0）
单纯肝脏	7（8.0）
皮肤+肠道	18（20.7）
皮肤+肝脏	9（10.4）
肠道+肝脏	11（12.6）
皮肤+肠道+肝脏	19（21.8）
累及器官数量	
1个	30（34.5）
2个	38（43.7）
3个	19（21.8）
aGVHD严重程度	
Ⅱ度	11（12.6）
Ⅲ度	55（63.2）
Ⅳ度	21（24.1）
明尼苏达危险度积分	
标危组	27（31.0）
高危组	60（69.0）
糖皮质激素治疗反应	
耐药	49（56.3）
依赖	38（43.7）
联合应用其他二线药物	
无	15（17.2）
是	72（82.8）

注：AML：急性髓系白血病；ALL：急性淋巴细胞白血病；CML：慢性髓性白血病；MDS：骨髓增生异常综合征；SAA：重型再生障碍性贫血；CR1：第1次完全缓解；CR2：第2次完全缓解；HCT-CI：造血干细胞移植合并症指数

2. 总体疗效：在巴利昔单抗治疗后第7、14、21、28、42、56天分别进行疗效评估。全部87例患者ORR为66.7％（58/87），35例（40.2％）获得CR，23例（26.4％）获得PR，29例患者疗效评估为NR。用药后第7、14、21、28、42、56天的ORR分别为16.1％（14/87）、42.5％（37/87）、58.6％（51/87）、58.6％（51/87）、65.5％（57/87）、66.7％（58/87）。巴利昔单抗治疗后中位起效（疗效评估达到≥PR）时间为12（4～44）d。经典型aGVHD组、迟发型aGVHD组ORR分别为77.8％（42/54）、48.5％（16/33）。单独应用巴利昔单抗治疗15例，巴利昔联合间充质干细胞治疗65例，联合芦可替尼治疗13例。单独应用巴利昔组患者整体病情较轻，其中明尼苏达危险度积分高危患者6例；联合用药组患者病情较重，其中明尼苏达危险度积分高危患者53例。单独应用巴利昔组ORR为86.7％（13/15），联合用药组ORR为62.5％（45/72）。不同aGVHD类型、累及器官数量及严重程度患者的治疗反应及起效时间见表2。

**表2:** 巴利昔单抗在不同类型糖皮质激素耐药/依赖急性移植物抗宿主病（aGVHD）患者中的疗效

组别	例数	CR［例（％）］	总反应［例（％）］	起效时间［d，*M*（范围）］
aGVHD类型				
经典型	54	29（53.7）	42（77.8）	11（4~44）
迟发型	33	6（33.3）	16（48.5）	13（7~36）
受累器官				
单纯皮肤	3	3（100.0）	3（100.0）	5（4~37）
单纯肠道	20	11（55.0）	15（75.0）	9（4~44）
单纯肝脏	7	3（42.9）	5（71.4）	6（4~42）
皮肤+肠道	18	7（38.9）	14（77.8）	13（5~32）
皮肤+肝脏	9	5（55.6）	8（88.9）	14（5~36）
肠道+肝脏	11	3（27.3）	4（36.4）	12（9~29）
皮肤+肠道+肝脏	19	4（21.1）	10（52.6）	15（10~19）
受累器官数量				
1个	30	16（53.3）	22（73.3）	8（4~44）
2个	38	15（39.5）	26（68.4）	13（5~36）
3个	19	4（21.1）	10（52.6）	15（10~19）
严重程度				
Ⅱ度	11	7（63.6）	10（91.0）	10（4~42）
Ⅲ度	55	21（38.2）	36（65.5）	11（4~44）
Ⅳ度	21	7（33.3）	11（52.4）	16（5~37）
明尼苏达危险度分组				
标危	27	17（63.0）	23（85.2）	11（4~44）
高危	60	18（30.0）	35（58.3）	13（4~33）

注：CR：完全缓解

根据患者年龄、性别、恶性血液疾病移植前状态、HCT-CI、造血干细胞来源、供受者性别关系等可能影响巴利昔单抗总反应率相关因素进行单因素分析。女性（*P*＝0.021）、haplo-HSCT（*P*＝0.012）、单个核细胞数小于中位数8.43×10^8^/kg（*P*＝0.034）、CD34^+^细胞数≥中位数3.12×10^6^/kg（*P*＝0.048）、明尼苏达积分标危（*P*＝0.014）、糖皮质激素依赖（*P*＝0.032）、经典型aGVHD（*P*＝0.005）、肝脏未受累（*P*＝0.033）、存在非GVHD所致重要脏器功能受损（*P*＝0.010）、合并活动性感染（*P*＝0.004）的患者巴利昔单抗治疗后ORR较高（表3）。

**表3:** 影响巴利昔单抗治疗急性移植物抗宿主病（aGVHD）总反应率（ORR）的单因素分析

影响因素	例数	ORR［％（例数）］	统计量	*P*值
年龄			0.025	0.875
≤40岁	55	67.3（37）		
>40岁	32	65.6（21）		
性别			5.331	0.021
男	51	56.9（29）		
女	36	80.6（29）		
恶性血液疾病移植前疾病状态			0.693	0.405
CR1	31	74.2（23）		
非CR1	43	65.1（28）		
HCT-CI分组			0.702	0.402
低危	62	69.4（43）		
中、高危	25	60.0（15）		
供者类型			8.869	0.012
同胞全相合	33	48.5（16）		
单倍型	50	76.0（38）		
造血干细胞来源			0.315	0.574
外周血	69	65.2（45）		
其他（外周血+骨髓、骨髓）	18	72.2（13）		
供受者性别关系			0.914	0.403
女供男	17	76.5（13）		
其他	70	64.3（45）		
供者、患者血型			2.314	0.128
相合	40	75.0（30）		
不合	47	59.6（28）		
回输单个核细胞数			4.506	0.034
≥中位数	43	55.8（24）		
<中位数	44	77.3（34）		
回输CD34^+^细胞数			3.898	0.048
≥中位数	46	76.1（35）		
<中位数	41	56.1（23）		
基线aGVHD严重程度			3.330	0.091
Ⅱ度	11	90.1（10）		
Ⅲ/Ⅳ度	76	63.1（48）		
明尼苏达危险度分组			6.024	0.014
标危	27	85.2（23）		
高危	60	58.3（35）		
甲泼尼龙剂量			0.099	0.753
1 mg·kg^−1^·d^−1^	55	65.5（36）		
2 mg·kg^−1^·d^−1^	32	68.8（22）		
糖皮质激素治疗反应			5.579	0.032
耐药	49	57.1（28）		
依赖	38	78.9（30）		
aGVHD类型			7.909	0.005
经典型	54	77.8（42）		
迟发型	33	48.5（16）		
用药前累及皮肤			1.145	0.285
是	49	71.4（35）		
否	38	60.5（23）		
用药前累及肝脏			4.521	0.033
是	46	56.5（26）		
否	41	78.0（32）		
用药前累及肠道			1.650	0.274
是	68	63.2（43）		
否	19	78.9（15）		
受累器官数			2.155	0.142
1～2个	68	70.6（48）		
3个	19	52.6（10）		
用药前合并活动性感染			8.488	0.004
是	37	83.8（31）		
否	50	54.0（27）		
合并非GVHD所致重要脏器功能受损			6.599	0.010
否	68	3.5（50）		
是	19	2.1（8）		
联合其他二线用药			2.266	0.132
无	15	6.7（13）		
是	72	2.5（45）		

注：CR1：第1次完全缓解；HCT-CI：造血干细胞移植合并症指数

3. 不良反应：用药过程中未观察到过敏、肝、肾功能损害。在23例（26.4％）患者中观察到不同程度血液学毒性（白细胞/血小板减少），1～4级血液学毒性发生率分别为4.6％（4/87）、12.6％（11/87）、5.7％（5/87）、3.4％（3/87）。

4. 感染并发症：用药后55例（63.2％）患者发生感染，43例（49.7％）发生病毒感染，26例（29.9％）发生细菌感染，6例（6.9％）发生血流感染，19例（21.8％）发生肺感染，5例（5.7％）发生真菌感染。巴利昔单抗有效组、无效组感染发生率分别为62.1％（36/58）、65.5％（19/29）（*χ*^2^＝0.099，*P*＝0.753）。35例患者发生巨细胞病毒（CMV）感染（CMV血症），中位CMV感染时间为用药后15（1～33）d。

5. 生存分析：本研究中位随访时间为154（4～1813）d。87例患者移植后6个月、1年OS率分别为44.8％（95％*CI* 39.5％～50.1％）、39.4％（95％*CI* 34.2％～44.3％），OS曲线见图1。

**图1:**
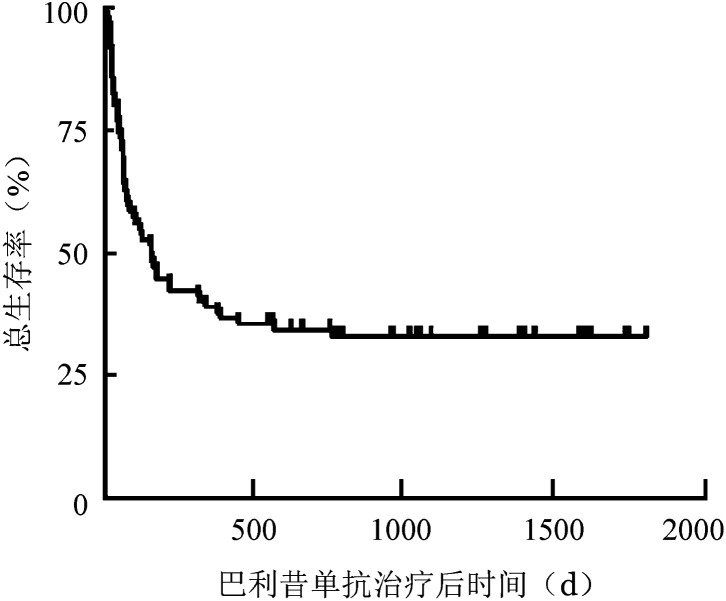
87例糖皮质激素耐药/依赖急性移植物抗宿主病患者巴利昔单抗治疗后总生存曲线

巴利昔单抗治疗后CR组（35例）、PR组（23例）、NR组（29例）移植后6个月OS率分别为80.0％（95％*CI* 73.2％～86.8％）、39.1％（95％*CI* 28.9％～49.3％）、6.9％（95％*CI* 2.2％～11.6％）（*χ*^2^＝34.679，*P*<0.001），移植后1年OS率分别为74.3％（95％*CI* 66.9％～81.7％）、30.4％（95％*CI* 20.8％～40.0％）、3.4％（95％*CI* 0％～6.8％）（*χ*^2^＝43.339，*P*<0.001）。OS曲线见图2。

**图2:**
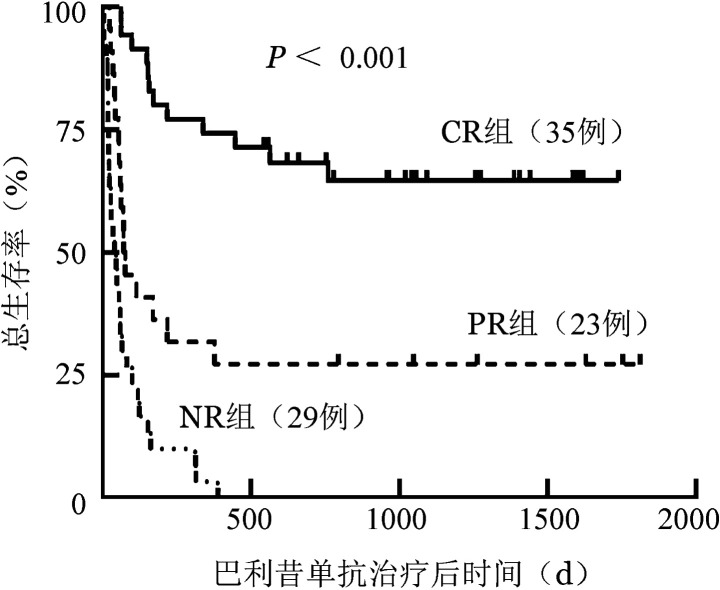
糖皮质激素耐药/依赖急性移植物抗宿主病巴利昔单抗不同疗效组总生存曲线 CR：巴利昔单抗治疗后完全缓解；PR：巴利昔单抗治疗后部分缓解；NR：巴利昔单抗治疗后未缓解

经典型aGVHD组、迟发型aGVHD组移植后6个月OS率分别为57.4％（95％*CI* 50.7％～64.1％）、24.2％（95％*CI* 16.7％～31.7％）（*χ*^2^＝9.109，*P*＝0.004），移植后1年OS率分别为51.9％（95％*CI* 45.1％～58.7％）、18.2％（95％*CI* 11.5％～24.9％）（*χ*^2^＝9.753，*P*＝0.003）。OS曲线见图3。

**图3:**
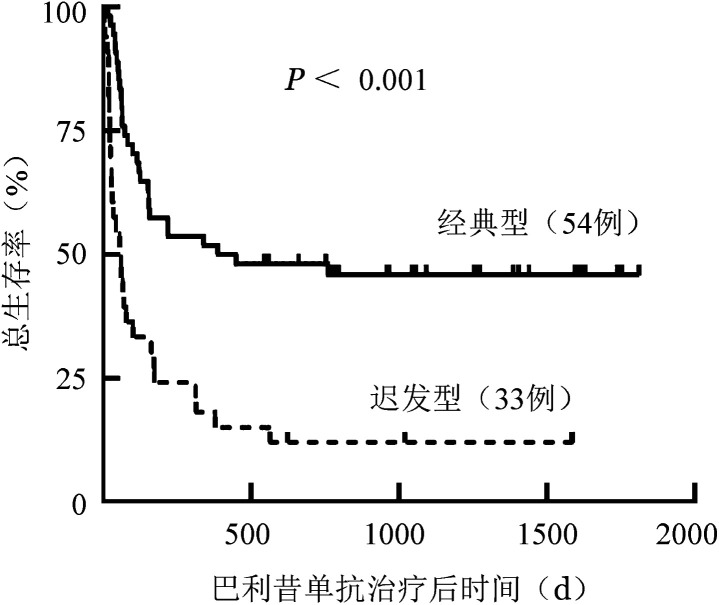
经典型、迟发型糖皮质激素耐药/依赖急性移植物抗宿主病巴利昔单抗治疗后总生存曲线

将患者性别、移植类型、回输干细胞数量等因素纳入生存分析，结果显示男性（*OR*＝0.432，95％*CI* 0.241～0.740，*P*＝0.003）、迟发型aGVHD（*OR*＝3.121，95％*CI* 1.770～5.503，*P*<0.001）、用药前明尼苏达积分高危组（*OR*＝3.591，95％*CI* 1.931～6.679，*P*<0.001）、用药前存在活动性感染（*OR*＝1.881，95％*CI* 1.029～3.438，*P*＝0.040）、伴有非GVHD所致重要脏器功能受损（*OR*＝3.100，95％*CI* 1.570～6.121，*P*＝0.001）是影响OS的独立危险因素（表4）。

**表4:** 糖皮质激素耐药/依赖急性移植物抗宿主病（aGVHD）患者巴利昔单抗治疗后总生存影响因素的分析结果

影响因素	单因素分析		多因素分析	
	*χ*^2^值	*P*值	*P*值	*OR*值（95%*CI*）
性别（男，女）	6.093	0.014	0.003	0.432（0.241~0.740）
移植类型（同胞全相合移植，单倍型移植）	0.340	0.560		
回输单个核细胞数（≥中位数，<中位数）	0.599	0.439		
回输CD34^+^细胞数（≥中位数，<中位数）	3.766	0.052		
用药前明尼苏达积分（标危，高危）	5.056	0.025	<0.001	3.591（1.931~6.679）
一线糖皮质激素治疗反应（耐药，依赖）	1.658	0.198		
aGVHD类型（经典型，迟发型）	16.989	<0.001	<0.001	3.121（1.770~5.503）
用药前aGVHD累及肝脏（是，否）	0.812	0.368		
用药前存在活动性感染（是，否）	7.906	0.005	0.040	1.881（1.029~3.438）
合并非GVHD所致重要脏器功能受损（否，是）	9.080	0.003	0.001	3.100（1.570~6.121）
联合其他二线用药（否，是）	1.250	0.264		
是否有血细胞减少（是，否）	0.080	0.778		

6. 复发及慢性移植物抗宿主病（cGVHD）：迟发型aGVHD组中部分患者合并cGVHD，故不纳入复发及cGVHD分析。经典型aGVHD组移植后共有5例急性白血病患者复发，3年累积复发率为（9.2±4.0）％，中位复发时间为115（54～448）d。移植后6个月、1年、3年非复发死亡率分别为（37.0±6.6）％、（40.7±3.6）％、（44.8±6.9）％。移植后1年、2年、3年cGVHD累积发生率分别为（2.9±2.9）％、（14.0±6.7）％、（38.4±9.9）％，中位发生时间为移植后211（92～387）d，均为轻、中度，累及皮肤、口腔、泪腺、肝、肺、肠道，其中以皮肤受累最为常见68.4％（13/19）。

## 讨论

巴利昔单抗是目前国内外治疗allo-HSCT后aGVHD的二线推荐治疗之一[1],[10]，国内外多项研究证实其对激素治疗失败aGVHD有较好疗效[3]–[6]。本组患者ORR为66.7％（58/87），其中CR率为40.2％（35/87），移植后6个月、1年OS率分别为44.8％（95％*CI* 39.5％～50.1％）、39.4％（95％*CI* 34.2％～44.3％），ORR及OS率均低于国内学者报道水平，这可能与本组病例多数患者原发病控制欠佳有关（恶性血液疾病CR1患者仅31例），也可能与将移植后复发患者供者淋巴细胞输注（DLI）后发生的aGVHD纳入研究相关。本研究中经典型aGVHD组ORR为77.8％（42/54）、移植后1年OS率分别为51.9％（95％*CI* 45.1％～58.7％），与国内外报道相接近；迟发型aGVHD组ORR为48.5％（16/33）、移植后1年OS率为18.2％（95％*CI* 11.5％～24.9％），两组差异有统计学意义（*P*＝0.005），这也间接证明虽然迟发型aGVHD某些临床症状与经典型aGVHD相似，但二者免疫背景、发病机制、原发疾病状态之间存在差异，不能机械按照经典型aGVHD诊疗路径处理。对于迟发型aGVHD患者而言，DLI输注后GVHD的充分预防以及发生aGVHD时早期积极干预可能会使患者更获益。

本研究未观察到巴利昔单抗严重药物过敏及输液反应。感染和不同程度的血液学毒性（白细胞/血小板减少）为最常见不良反应。用药后发生感染发生率为63.2％（55/87），病毒感染为最常见（43/87，49.7％），与文献[4]–[5]报道的结果相接近。移植后血细胞减少可能由病毒感染、GVHD、药物、复发等多种原因引起，笔者记录了巴利昔单抗用药期间所有血细胞减少病例除外使用可能引起血细胞减少的药物患者，用药期间不同程度的血细胞减少发生率为25.3％（22/87），3～4级血液学毒性发生率为9.1％（8/87），而国内学者研究中却未观察到明显血液学毒性[4]–[5]，这可能与本研究观察对象并非均为经典型aGVHD病例相关。本研究样本量较小，血细胞减少是否影响OS有待扩大样本量进一步分析。

既往研究显示巴利昔单抗治疗不同部位aGVHD的疗效不同，皮肤型最好，肝脏型最差[4]–[6]。本研究单纯累及皮肤患者ORR为100.0％（3/3），单纯累及肝脏患者ORR为71.4％（5/7），单纯累积肠道患者ORR为75.0％（15/20），但因样本量较小，不同部位受累的疗效有待进一步研究。刘文宾等[9]报道，在haplo-HSCT模式下移植物中单个核细胞数>8.33×10^8^/kg是发生糖皮质激素耐药GVHD的独立高危因素。本研究中单个核细胞数<中位数组（<8.43×10^8^/kg）ORR高于≥中位数组（*P*＝0.034），糖皮质激素依赖组ORR高于糖皮质激素耐药组（*P*＝0.032），提示移植物中单个核细胞数量可作为巴利昔单抗治疗反应的预测指标。

本研究单因素及多因素分析均提示巴利昔单抗用药前存在活动性感染（*OR*＝1.881, 95％*CI* 1.029～3.438，*P*＝0.040）、非GVHD所致重要脏器功能受损（*OR*＝3.100，95％*CI* 1.570～6.121，*P*＝0.001）是影响巴利昔单抗疗效的独立危险因素，提示对于移植后存在感染及重要脏器功能障碍的患者，aGVHD的预防更应得到重视。

既往研究认为男性患者与女性供者组合是发生aGVHD的高危因素[1]，在本组病例中女供男移植占19.5％（17/87），女供男组ORR为76.5％（13/17），非女供男组ORR为64.3％（45/70），两组间差异无统计学意义（*P*＝0.403）。本次研究也观察到男性患者ORR及OS均低于女性患者，这与国内学者以往报道的结果[5]–[6]存在一定分歧。因本研究病例相对较少，关于巴利昔单抗治疗aGVHD的疗效是否受到供、受者性别影响尚有待进一步研究。

本研究明尼苏达危险度积分标危组与高危组OS率具有显著性差异（*OR*＝3.591，95％*CI* 1.931～6.679，*P*<0.001），这与Liu等[6]报道的结果相一致。本组病例巴利昔单抗治疗后用药后第7、14、21、28、42、56天的ORR分别为16.1％（14/87）、42.5％（37/87）、58.6％（51/87）、58.6％（51/87）、65.5％（57/87）、66.7％（58/87），即大多数患者在治疗在使用4剂巴利昔单抗可观察到疗效。因此，明尼苏达高危、巴利昔单抗治疗早期反应不佳均提示预后不良，对于这类患者需及时干预。

目前国内外糖皮质激素耐药依赖aGVHD二线治疗仍在不断探索中。Tan等[11]联合应用巴利昔单抗和依那西普治疗重度（Ⅲ/Ⅳ度）糖皮质激素耐药/依赖aGVHD患者，与经典挽救治疗相比，巴利昔单抗和依那西普联合治疗可提高内脏aGVHD的CR率，并显著提高2年OS率（54.7％对14.8％，*P*<0.001）。Nadeau等[12]将巴利昔单抗联合英夫利昔单抗治疗Ⅲ/Ⅳ级肠道aGVHD却并未观察到获益。国内学者采用脐血间充质干细胞预防及治疗aGVHD也取得了一定的疗效[13]–[14]。芦可替尼治疗糖皮质激素耐药/依赖aGVHD也被证实是一项安全有效的治疗选择[15]–[17]。本研究中Ⅲ/Ⅳ度患者占87.4％（76/87），有82.8％（72/87）的患者选用一种或者两种二线药物联合治疗aGVHD（包括间充质干细胞、芦可替尼、依那西普等），可能由于患者病情较重，样本量相对较小，笔者未观察到其他二线药物对巴利昔单抗治疗ORR及OS的影响（*P*>0.05）。但从目前研究来看，不同二线药物对不同类型GVHD具有不同优势，对于多器官受累的重度aGVHD患者，探索不同二线药物联用尽早控制病情仍是今后需要不断研究的方向。

总而言之，本研究再次证实了巴利昔单抗治疗糖皮质激素耐药/依赖aGVHD的安全性及有效性，但通过对病例资料进行单因素、多因素分析发现，迟发型、用药前明尼苏达积分高危组、存在感染或重要脏器功能受损患者疗效不佳，在治疗前对存在上述高危因素的患者应该及时作出相应干预。
